# Genotype by environmental interactions shape insecticide resistance phenotypes in *Culex pipiens* and *Culex restuans*

**DOI:** 10.1038/s41437-026-00833-w

**Published:** 2026-03-07

**Authors:** Kylee R. Noel, Carly Kallembach, Mahica Iyer, Carla E. Cáceres, Chris M. Stone

**Affiliations:** 1 Department of Entomology, School of Integrative Biology, University of Illinois Urbana-Champaign, Champaign, IL USA; 2 Illinois Natural History Survey, Prairie Research Institute, University of Illinois Urbana-Champaign, Champaign, IL USA; 3 Department of Evolution, Ecology, and Behavior, School of Integrative Biology, University of Illinois Urbana-Champaign, Champaign, IL USA

**Keywords:** Genetic variation, Evolutionary ecology

## Abstract

Anthropogenic changes can drive rapid evolution in wild populations, but the role of phenotypic plasticity in such scenarios remains unclear. This uncertainty can affect applications like the design of resistance management approaches. In the case of insecticide resistance in mosquitoes, however, little is known regarding how environmental conditions, genetic variation, and their interactions jointly shape resistance phenotypes. To address this, we employed a full-sibling design to investigate the effects of larval food availability on adult broad-sense heritability and phenotypic plasticity in resistance to permethrin. Two experiments measured resistance levels in West Nile virus vectors (laboratory colony of *Culex pipiens* and two field populations of *Culex restuans*) using CDC bottle bioassays, and the time until death was tracked. Wing lengths were measured to assess if there is a relationship between body size and permethrin resistance. Based on likelihood ratio tests, the broad-sense heritability values for resistance were significant. There was substantial variance and phenotypic plasticity in both *Cx. restuans* field populations, while the laboratory colony of *Cx. pipiens* exhibited less variation. Larval food availability significantly affected resistance, but the sign of the effect varied across populations from different geographic regions, highlighting the importance of genotype by environmental interactions in this system. Our results offer valuable insights into the potential for insecticide resistance to evolve in mosquito populations and have important implications for how resistance in vectors can be assessed. We suggest changes to improve the current methodology for insecticide resistance testing and recommend that population-specific data should inform vector control schemes.

## Introduction

Since the 1800s, humans have been global-scale drivers of environmental changes (Steffen et al. 2011), which drive rapid evolution in wild populations. These anthropogenic changes, including habitat modification, pollution, and climate change, have majorly impacted global biodiversity, including driving rapid evolution (Pelletier and Coltman 2018; Jørgensen et al. 2019; Schell et al. 2021) and these impacts are projected to increase in the future (Powers and Jetz 2019; Yang et al. 2025). Given this, wild populations increasingly need to respond to novel environmental conditions to persist, and the extent to which they can handle these changes remains under question (Pelletier and Coltman 2018; Catullo et al. 2019).

Organisms can respond in different ways to rapid environmental changes, with the quickest responses being relocation and phenotypic plasticity (Pelletier and Coltman 2018). These methods do not necessarily lead to rapid evolution. In fact, plasticity may shield organisms from evolutionary change (Fox et al. 2018; Catullo et al. 2019). On the other hand, plasticity can facilitate evolutionary responses by decreasing mortality rates and sustaining population viability during rapid environmental changes, thus allowing more time for organisms to adapt to selection pressures (Chevin and Hoffmann 2017; Catullo et al. 2019). The degree to which populations can respond to selection is determined by the trait’s broad-sense heritability, the ratio of genetic to phenotypic variance, which is specific to a given population in a given environment (Firko and Hayes 1990; Falconer 1996). Understanding how populations respond to environmental changes will help us to better predict and potentially manage outcomes for at-risk populations.

A well-known example of rapid evolution is the development of resistance to pesticides and herbicides (Ffrench-Constant 2013; Liu 2015; Garnas 2018; Reznick et al. 2019). Pyrethroids are synthetic organic insecticides with low mammalian toxicity and are used in agricultural and urban pest control worldwide, and have been widely detected in a range of environments (Tang et al. 2018; Stehle et al. 2019). In the environment, pyrethroids have been shown to have enhanced or reduced toxicity when combined with heavy metals (Wang et al. 2019), or with changes in temperature (Grafius 1986; Hodjati and Curtis 1999), and are rapidly degraded because of high sensitivity to light, pH, temperature, and moisture (Meena et al. 2022). In short, the fact that pesticide toxicity is determined by other environmental conditions complicates our understanding of the evolution of resistance.

How environmental conditions, phenotypic plasticity, and genetic variation jointly shape the expression and selection for insecticide resistance is not well understood for many of the populations being targeted by insecticides. Extrapolation from laboratory experiments to field conditions is challenging because natural environments vary widely in resource availability, density, and microbial composition, all of which can induce plastic changes in physiology and life history traits that alter the apparent expression and fitness cost of resistance (Kulma et al. 2013; Nkya et al. 2013). For example, nutrition and age of exposure during the larval stage have been shown to increase insecticide resistance in the adult stage of the mosquito *Culex quinquefasciatus* (Ong and Jaal 2018). Changes in phenotypic resistance have also been shown to occur in response to differences in larval environment in *Anopheles* species, including changes in temperature, population density, and nutrition (Kulma et al. 2013; Owusu et al. 2017). These studies show that environmental factors affecting body size and resource availability during the larval stages can carry over and change the resistance phenotype of the adult stage, with larger mosquitoes being more likely to survive insecticide exposure (Kulma et al. 2013; Owusu et al. 2017; Ong and Jaal 2018). Thus, the resistant phenotype may be much more variable under natural conditions than laboratory assays indicate, which can impact the outcomes of vector control measures and potentially have implications for the evolution of resistance itself.

With their large population sizes and fast generation times, mosquitoes provide a compelling system for exploring the role of phenotypic plasticity and rapid evolution (Wilke et al. 2014; Liao et al. 2024). Their importance as vectors of pathogens has made them a persistent target for insecticide intervention (Richards et al. 2020), and the resulting widespread resistance to pyrethroids has been reported in many countries (Collins et al. 2019; Khan 2020; Talipouo et al. 2021). A variety of selective pressures have been implicated in pyrethroid resistance in mosquitoes including insecticide use for personal protection, use against urban and agricultural pests, and the presence of other chemicals in the environment (Nkya et al. 2013; Tabbabi et al. 2019; Lee et al. 2023; Arich et al. 2024). While pyrethroids are typically used to target adult mosquitoes, these insecticides make their way into larval habitats via stormwater runoff and spray drift (Stehle et al. 2019).

West Nile virus (WNV) is the leading cause of mosquito-borne disease in the continental United States, with roughly 2000 reported human cases each year (CDC 2025). WNV is maintained in enzootic cycles between *Culex* mosquitoes (Diptera: Culicidae) and birds. *Culex* mosquitoes, though primarily ornithophilic in their nature, will also opportunistically feed on mammals, making these mosquitoes the link to human infections (O’Leary et al. 2004; Kilpatrick 2011). *Culex restuans* is a mosquito endemic to North America and is an early season vector of WNV in the midwestern and northeastern US (Johnson et al. 2015; Durden et al. 2025). Ecologically similar, North American subspecies of the *Culex pipiens* complex, which includes *Cx. pipiens pipiens* and *Cx. pipiens quinquefasciatus*, are also important vectors of WNV (Farajollahi et al. 2011). These subspecies are known to hybridize in a zone within the middle latitudes of the United States, and diapausing of adult *Cx. pipiens pipiens* infected with WNV provides an overwintering mechanism for the virus in northern latitudes (Kothera et al. 2009). The transmission of WNV can be reduced by managing mosquito populations with insecticides that target adult mosquitoes, known as adulticides (Reisen and Brault 2007), though these chemicals are also found in water sources globally (Stehle et al. 2018, 2019). *Culex* mosquitoes are typically container breeders, often found in human-made constructions (e.g., storm water ditches, construction sites, and tires) (Durden et al. 2025), and cannot avoid insecticide use by spatial movement. Populations of *Culex* mosquitoes have displayed varying levels of resistance to the most common classes of insecticides used in vector control (Dubie et al. 2022; Noel et al. 2025).

In this study, we performed two experiments, one using *Culex pipiens pipiens* (hereafter *Cx. pipiens*) from a laboratory colony and one using two separate field populations of *Culex restuans* mosquitoes to determine the effects of different environmental conditions on the broad-sense heritability of insecticide resistance using a full-sibling design. We explore the expression of plasticity in phenotypic insecticide resistance and whether the reaction norms vary among different populations. We used variation in larval food availability as our environmental parameter as it can vary drastically under field conditions in response to rainfall, seasonally, by habitat, or with mosquito density, and could affect resistance phenotypes either directly (e.g., through variation in expression of detoxification enzymes) or indirectly (through variation in body size which could, for instance, affect cuticle thickness and insecticide penetration) (Lease and Wolf 2010; Balabanidou et al. 2018). We hypothesize that the phenotypic expression of insecticide resistance in these mosquito species will be determined by a combination of environmental and genetic effects. If phenotypic plasticity in resistance is largely a consequence of variation in body size, we expect that the reaction norms will be similar between populations. A significant environmental component to resistance phenotypes will have implications for applied outcomes such as the monitoring and management of insecticide resistance.

## Materials and methods

A full-sibling design was used to evaluate the effects of larval diet on adult susceptibility to permethrin. Female *Culex* mosquitoes lay their eggs in rafts, which are clusters of eggs deposited on the water surface by a single female. This provides an opportunity to easily obtain full-sibling families (Mpho et al. 2002), as under field conditions females are typically monogamous and multiple insemination has been shown to occur infrequently (Kitzmiller and Laven 1958; Craig 1967). As *Cx. restuans* has never been successfully established into a laboratory colony, egg rafts for this experiment were collected directly from the field from two locations in Illinois (IL), and the larvae were placed into experimental conditions. GPS coordinates for these sampling locations are provided in the supplementary information (Table S1). *Culex pipiens* egg rafts were collected from four locations in southern IL in August 2022 and were combined for the establishment of a laboratory colony. Individuals from the F11 generation were used for the *Cx. pipiens* experiment. Since southern IL falls within the *Cx. p. pipiens* and *Cx. p. quinquefasciatus* hybridization zone, identifications were performed at the first larval instar to determine the species (Reiter 1986; Reiskind and Wilson 2008). The GPS coordinates for these sampling locations are listed in Table S2.

The amounts of food for the experimental treatment groups were determined from trials using the *Cx. pipiens* colony. The *Cx. pipiens* colony was maintained using a modified standard colony routine provided by the Centers for Disease Control and Prevention (CDC) (personal communication with Sean Masters, Insectary and Animal Care for the Division of Vector-Borne Diseases, CDC, Fort Collins, Co. 80521). Briefly, the colony larvae are reared at a density of 200 individuals per pan of 1.5 L of deionized (DI) water and provided with a larval diet equal to ~4 mg of total diet per larva. This feeding regime served as the basis to determine the experimental stressful conditions for the low food treatment group and overfeeding conditions for the high food treatment group for the experimental procedures.

Feeding regimes differed between the two experiments and are described in detail in the experimental procedure sections for each species below. For the *Cx. pipiens* experiment, the feeding regime was close to what the colony mosquitoes experienced during regular colony maintenance. The size of field-collected egg rafts can be affected by environmental conditions (Madder et al. 1983; Townroe and Callaghan 2015), and there was a greater degree of variation in *Cx. restuans* egg raft size compared with the *Cx. pipiens* from the colony. Given this, the amounts *Cx. restuans* were fed were determined using a per-individual calculation. The *Cx. restuans* were also fed in gradually increasing increments, as there was concern that providing lump sums of food in a similar way to the *Cx. pipiens* would have led to toxicity from excessive bacterial growth and larval mortality for trays set up with smaller egg rafts, or a greater degree of competition for those set up with larger egg rafts.

While the feeding regimes for each experiment are different, the following parameters remained constant for both. The larval diet consisted of TetraMin® (Tetra Holding (US)), brewer’s yeast (MP Biomedicals LLC), and rabbit chow (Kaytee Products, Inc.) in a 1:1:1 mixture. Rearing pans were kept in walk-in environmental chambers (Widman Mfg. Co., St. Paul, Minn. USA) at a temperature of 26 ± 1 °C, with a relative humidity of 70 ± 8%, and a photoperiod of 16 h light and 8 h dark. Pupae were transferred to cups of DI water and placed into adult cages (5.68-liter paper bucket cages) for emergence. Adults were provided with access to water and 10% honey solution.

### Experimental procedure - *Culex pipiens*

Mosquitoes from the F11 generation of the *Cx. pipiens* colony were used for this portion of the experiment, performed in August 2023. A single, unmated adult male was placed in a 5.68-liter paper bucket cage with five females to produce the families. A total of 20 cages were set up in this manner. Cages were contained in environmental chambers under the same insectary conditions described previously (26 ± 1 °C, 70 ± 8% relative humidity, and 16:8 h light/dark photoperiod). After 7 days, mosquitoes were given access to chicken blood in Alsever’s solution for 2 h using a Hemotek feeding system (Hemotek Ltd., Blackburn, UK). A day after blood feeding, an oviposition cup (volume 0.27 L) filled with grass infusion, prepared by steeping 25 g grass in 1 L of water (Lampman and Novak 1996), was provided for egg laying, and only one egg raft was collected from each cage. This produced 15 families in total. The larvae from each family were divided equally between a low or a high food treatment group, with one pan for each treatment per family. Families had an average of 48.69 ± 18.68 (mean ± SD) larvae per pan. High food treatment groups received 120 mg of diet on days 0, 2, 4, 5, 6, and 7, and low food treatment groups were provided 60 mg on those same days. This resulted in 720 mg of total diet for the high food treatment group pans and 360 mg of total diet for the low food treatment group pans.

### Experimental procedure - *Culex restuans*

Egg rafts were collected from two sampling locations, one centrally in Urbana, IL, and one further south in Metropolis, IL, during the summer of 2021. Collections were performed using 18.9-liter bins filled with 7.6 liters of grass infusion, which were set out overnight to attract gravid *Culex* mosquitoes to lay eggs. Bins were checked the following day for the presence of egg rafts. A small paintbrush was used to gently lift the rafts out of the grass infusion, and they were then placed into 12-well tissue culture plates filled with DI water for transportation back to the lab. Each egg raft was individually placed into a well, and identifications were performed at the first larval instar to determine the species (Reiter 1986; Reiskind and Wilson 2008). This resulted in 13 families from Urbana (central IL), and 15 from Metropolis (southern IL). The larvae from each family were divided equally between a low or a high food treatment group, with one pan for each treatment per family. The smallest family from southern IL had 62 larvae per treatment, and the largest had 120 larvae per treatment. From central IL, family sizes ranged from 64 to 123 larvae. Due to the large range in family sizes, food was provided based on a per-individual calculation. The low food treatment groups were provided with 3 mg of diet per larva and 6 mg of diet per larva for the high food treatments. The resulting range of diet provided to a pan was 186 mg as the lowest amount fed to the low food treatment group and 738 mg as the highest amount fed to the high food treatment group. Food was given in increments of 10% of the diet total on days 0, 2, 4, and 5, and 20% on days 6, 7, and 8.

### Insecticide resistance bioassays

Adults were kept in 5.68-liter paper bucket cages in environmental chambers under standard insectary conditions described previously (26 ± 1 °C, 70 ± 8% relative humidity, and 16:8 h light/dark photoperiod) and provided with a 10% honey solution. The resistance status of female mosquitoes aged 3–5 days was tested using modified CDC bottle bioassays (McAllister and Scott 2020) performed under the same environmental conditions. *Culex restuans* has never been established into a laboratory colony so there is no susceptibility information available for this species. Therefore, to assess the resistance levels in *Cx. restuans*, the diagnostic dose (43 mg/L permethrin) and diagnostic time (30 min.) for *Cx. pipiens* were used in accordance with the CDC’s guidelines (McAllister and Scott 2020; Noel et al. 2025). The ratio of surviving mosquitoes at that time is used to determine resistance levels. An average across experimental bottles of less than 90% mortality at the diagnostic time is considered indicative of resistance to that insecticide (McAllister and Scott 2020). Assays used 250 ml Wheaton bottles, coated with 1 ml of permethrin mixed with acetone for a final concentration of 43 mg/L, and a control bottle coated with 1 ml of acetone alone. Ten to fifteen females were introduced into bottles and were observed for 2 h, tracking mortality every five minutes. All adult females of the proper age were used, and one to two bottles were run for each family x treatment combination. Assay results were discarded if mortality in the control bottles was above 10%. Time until death was used as the response variable, indicating the extent of insecticide resistance in the analysis. After completion of the assay, the tested mosquitoes were stored at −80 °C in individual 1.5 ml tubes for wing dissections.

For the *Cx. pipiens* experiment, bioassays were performed on 403 females from 15 families (average 13.53 females ±1.2 SE per family x treatment combination). For the *Cx. restuans*, bioassays were performed on 558 females from 13 families (average 29.03 females ±1.2 SE per family x treatment combination) collected from the field in central IL. Bioassays for *Cx. restuans* from southern IL were performed on 773 females from 15 families (average 27.62 females ±2.1 SE per family x treatment combination).

### Wing lengths

Wing length is a trait that is expected to change depending on the food treatment group, as larval nutrients have been shown to have a significant effect on wing length (Alto et al. 2012). Wing length was assessed as a proxy for overall body size, as body size is a trait that has also been correlated to several important ecological and epidemiological factors, including fecundity (Mackay et al. 2023). Additionally, insecticide resistance has been associated with fitness costs such as a reduction in body size (Carriere et al. 1994), but it has also been associated with increased wing length per body size, which would provide several fitness advantages (e.g., increased flight ability, fecundity, and blood-feeding success) (Nasci 1987; Chan and Zairi 2013). When possible, both wings were removed from each female tested in the resistance bioassays, and the average lengths were used for analysis. Standard wing length (mm) was measured as the linear distance from the apical notch to the axillary margin, excluding the wing fringe for each mounted wing (Nasci 1987; Mohammed and Chadee 2011). Measurements were taken using an Olympus IX51 inverted microscope with the imaging software cellSens Entry (Evident Scientific, Inc.).

### Genotype × environmental effects

Genotype × environmental (G×E) effects on wing length and insecticide resistance were each tested using the two-way mixed model analysis of variance with food treatment, or ‘environment’ as a fixed factor and familial origin or ‘genotype’ as a random factor. The analyses were performed separately for both species. *Culex restuans* from both sampling locations were analyzed together with ‘location’ as an additional fixed factor.

### Heritability estimates

Broad-sense heritability of wing length and insecticide resistance were estimated using a random effects linear model (Ye et al. 2016). The family term was fitted as a random effect, and its significance was tested using likelihood ratio tests (LRT). The log-likelihood of the full linear model was compared to the log-likelihood of a reduced model that did not include the family term. Two times the difference in these log-likelihoods was tested against a chi-squared distribution with a single degree of freedom. Broad-sense heritability, H^*2*^, was estimated as two times the family variance component divided by the total phenotypic variance (Young et al. 1995). Given the experimental design (full-sibling analysis), heritability estimates in this study could be inflated due to common environment, maternal and dominance effects, and therefore should be considered as upper limits to the heritability (Falconer 1996).

### Data analysis

All data analyses were performed using R version 4.4.1 (R Core Team 2024). The residuals were log-transformed to normality for analysis using time until death from the insecticide resistance assays as the response variable. The models for G×E effects were fit and analyzed using the package ‘*lme4*’ (Bates et al. 2015). The significance of the environment was determined using an F-test, and log-likelihood ratio tests were used to determine the random effects of genotype and the interaction between genotype and environment. Log-likelihood ratio tests were performed using the ‘*anova’* function included in the base R ‘*stats*’ package. Variance components for random effects were extracted using the ‘*VarCorr*’ function from the ‘*lme4*’ package (Bates et al. 2015). A *post hoc* pairwise test of means using the Tukey method (package ‘*emmeans*’) (Lenth et al. 2024) was used to compare the *Cx. restuans* results between locations. We analyzed the effects of wing length on insecticide resistance using a linear model with the time of death as the response variable and wing length as the explanatory variable, which was assessed using the ‘*ANOVA’* procedure within the package ‘*car’*, using a type III hypothesis (Fox et al. 2001).

## Results

### *Culex pipiens*

**Fig. 1:**
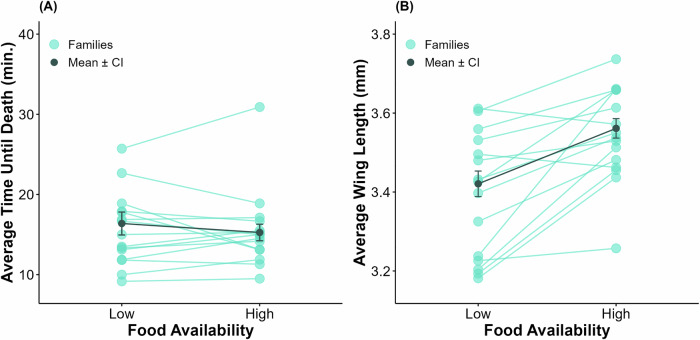
Effects of food availability on insecticide resistance and wing length of *Culex pipiens.* Genotype means of **A** average time until death and **B** wing lengths for *Cx. pipiens* (*N* = 15 families). The light green points represent the genotype’s average, and lines connecting points across diets represent the same genotype. The dark gray points represent the overall average for each treatment with 95% confidence intervals.

One hundred percent mortality was observed by the end of the scoring period for all bottles in this experiment. Genotype influenced insecticide resistance (LRT: *D* = 12.76, *p* < 0.001), but there was no significant effect of food treatment (*F*_*1,9.98*_ = 0.17, *p* = 0.687) or the interaction of genotype and food treatment (LRT: *D* = 0.17, *p* = 0.97) (Fig. 1A). The environmental means for the low and high food treatment groups did not significantly differ (Table 1), though the reaction norms show genotype differences for insecticide resistance (Fig. 1A). Increases in insecticide resistance from the low to the high food treatment group are shown in 47% of families, 40% of families decreased, and 13% stayed the same between environments. The broad-sense heritability (H^2^) of resistance for the low food treatment group was estimated to be 0.44 (LRT: *D* = 18.14, *p* < 0.001) and 0.49 (LRT: *D* = 24.41, *p* < 0.001) for the high food treatment group. These similar heritability estimates for both environments were reflected in the similarity in response to insecticide between the two food treatment groups.

**Table 1:** Results from CDC bottle bioassays (time until death (min.)) testing permethrin (43 mg/L) averaged by population and associated broad-sense heritability estimates (H^2^).

Food Availability	Population	Species	Mean	SD	H^2^
Low	Colony	*Cx. pipiens*	15.26	7.92	0.44
	Central	*Cx. restuans*	32.79	23.30	0.85
	Southern	*Cx. restuans*	44.32	22.67	0.52
High	Colony	*Cx. pipiens*	16.38	9.55	0.49
	Central	*Cx. restuans*	62.99	34.91	0.48
	Southern	*Cx. restuans*	36.00	26.36	0.75

Wing length was influenced by food treatment (*F*_*1,13.37*_ = 22.11, *p* < 0.001), genotype (LRT: *D* = 5.9, *p* = 0.016), and their interaction (LRT: *D* = 15.3, *p* < 0.001) (Fig. 1B). On average, individuals in the high food treatment group were larger than those in the low food treatment group (Table 2). The degree of change in average wing length between food treatments varied considerably; some families experienced substantial increases in size, while few remained relatively stable, and two families had a slight decrease in size in the high food treatment group. H^2^ for the wing length of the low food treatment group was estimated as 0.93 (LRT: *D* = 68.40, *p* < 0.001) and H^2^ for the wing length of the high food treatment group was 0.65 (LRT: *D* = 55.01, *p* < 0.001). Body size was not found to have a significant effect on insecticide resistance (*F*_*1,28*_ = 0.54, *p* = 0.469). There was concern that raising the mosquitoes in different larval densities, due to variation in egg raft size, affected the availability of the diet and thus the overall size of the mosquitoes in these experiments, particularly for the *Cx. pipiens* that were not fed on a per-larva basis. To examine this, we used a linear model with average family wing length as the response variable and total number of larvae as the explanatory variable, and found no relationship between the two (*F*_*1,84*_ = 0.006, *p* = 0.9383).

**Table 2:** Average wing length measurements (mm) by population and associated broad-sense heritability estimates (H^2^).

Food Availability	Population	Species	Mean	SD	H^2^
Low	Colony	*Cx. pipiens*	3.42	0.21	0.93
	Central	*Cx. restuans*	3.34	0.23	0.99
	Southern	*Cx. restuans*	3.31	0.17	0.87
High	Colony	*Cx. pipiens*	3.56	0.19	0.65
	Central	*Cx. restuans*	3.48	0.18	0.89
	Southern	*Cx. restuans*	3.44	0.15	0.85

### *Culex restuans*

**Fig. 2:**
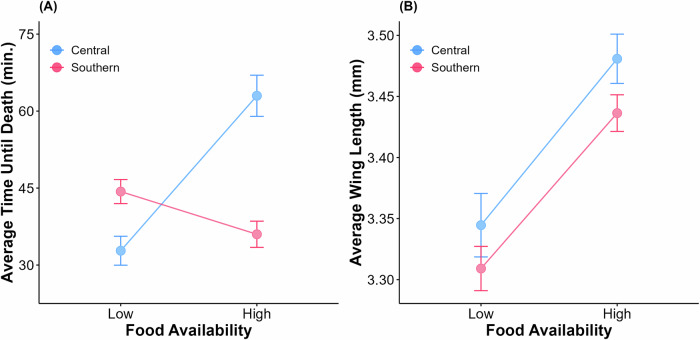
Comparison of the central and southern IL *Culex restuans* field populations. **A** Population averages and 95% confidence intervals for insecticide resistance testing and **B** wing length measurements for the central IL (blue) and southern IL (pink) *Cx. restuans*.

**Fig. 3:**
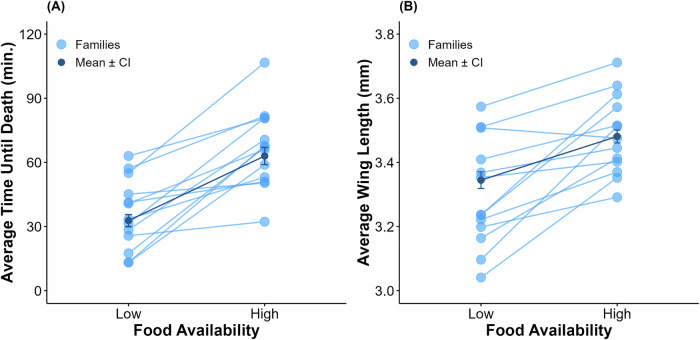
Effects of food availability on insecticide resistance and wing length of *Culex restuans* from central IL. Genotype means of **A** average time until death and **B** wing lengths for the *Cx. restuans* collected from central IL (*N* = 13 families). The light blue points represent the genotype’s average, and lines connecting points across diets represent the same genotype. The dark blue points represent the overall average for each treatment with 95% confidence intervals.

**Fig. 4:**
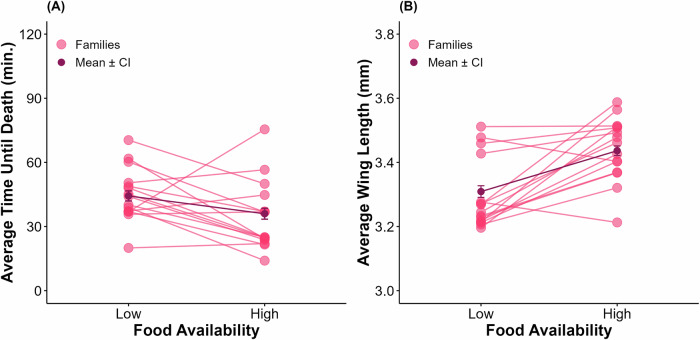
Effects of food availability on insecticide resistance and wing length of *Culex restuans* from southern IL. Genotype means of **A** average time until death and **B** wing lengths for the *Cx. restuans* collected from southern IL (*N* = 15 families). The light pink points represent the genotype’s average, and lines connecting points across diets represent the same genotype. The dark pink points represent the overall average for each treatment with 95% confidence intervals.

One hundred percent mortality was observed by the end of the scoring period for all bottles in this experiment. Insecticide resistance was influenced by the effects of food treatment (*F*_1,26.73_ = 4.54, *p* = 0.042), the interaction between food treatment and genotype (LRT: *D* = 152.63, *p* < 0.001), and the interaction between food treatment and collection location (*F*_1,26.73_ = 34.82, *p* < 0.001), but there were no significant effects of location (*p* = 0.187) or genotype (*p* = 0.141) alone (Fig. 2A). A post hoc Tukey test for multiple comparisons found that the mean value of insecticide resistance was significantly different between the central low and the central high food treatment groups (*p* < 0.001), the central high and the southern high food treatment groups (*p* < 0.001), and the southern low and the southern high food treatment groups (*p* = 0.045) (Fig. 2A). On average, individuals collected from central IL displayed higher resistance in the high food treatment group compared with the low (Table 1). Every family experienced an increase in resistance from the low to high food treatment groups, but the degree of increase varied among families; the lowest was an increase of 5.24 min. and the highest was 54.51 min. (Fig. 3A). The opposite is true for southern IL, where mosquitoes collected from this location showed a higher average resistance level in the low food treatment group compared to the high (Table 1). From this location, 33% of families displayed an increase in resistance from low to high food treatment and 67% of families experienced a decrease (Fig. 4A). H^2^ of resistance for the central IL low food group was estimated to be 0.85 (LRT: *D* = 112.11, *p* < 0.001) and 0.48 (LRT: *D* = 61.99, *p* < 0.001) for the high food treatment group. Mosquitoes from this population had a higher H^2^ value for the low food treatment group than the high group. H^2^ of resistance for the southern low food treatment group was estimated to be 0.52 (LRT: *D* = 51.24, *p* < 0.001) and 0.75 (LRT: *D* = 137.22, *p* < 0.001) for the high food group.

Wing lengths for *Cx. restuans* were influenced by food treatment (*F*_1,25.56_ = 40.34, *p* < 0.001), genotype (LRT: *D* = 5.54, *p* = 0.019), and the interaction between treatment and genotype (LRT: *D* = 174.26, *p* < 0.001), but there was no effect of sampling location (*p* = 0.530) (Fig. 2B). A post hoc Tukey test for multiple comparisons found that the mean value of insecticide resistance was significantly different between the central low and the central high food treatment groups (*p* < 0.001), the central low and the southern high food treatment groups (*p* = 0.034), the central high and the southern low food treatment groups (*p* = 0.002), and the southern low and the southern high food treatment groups (*p* = 0.002) (Fig. 2B). On average, mosquitoes from both the central and southern IL locations had larger wing sizes in the high food treatment group compared to the low (Table 2). One family from central IL displayed a decrease in average wing length from low to high food treatments, while the rest showed increases ranging from 0.05 to 0.42 mm (Fig. 3B). From southern IL, most families also showed increases in average wing length going from the low to the high food treatment group with average increases ranging from 0.05 to 0.35 mm, one family stayed stable between the two environments, and two families decreased in average wing length (Fig. 4B). H^2^ was estimated to be 0.99 (LRT: *D* = 170.87, *p* < 0.001) for the central IL low food treatment group and 0.89 (LRT: *D* = 130.34, *p* < 0.001) for the high food treatment group. Body size was found to have a significant effect on insecticide resistance (*F*_1,24_ = 7.69, *p* = 0.01). H^2^ for wing length of the southern low food treatment group was estimated to be 0.87 (LRT: *D* = 158.24, *p* < 0.001) and H^2^ for wing length of the high food treatment group was 0.85 (LRT: *D* = 164.31, *p* < 0.001). In contrast to the central IL population, here body size was not found to have a significant effect on insecticide resistance (*F*_1,28_ = 2.41, *p* = 0.132).

## Discussion

There were regional differences in insecticide resistance among the sampled *Cx. restuans* populations from the field, while the *Cx. pipiens* from the colony showed no resistance in either food treatment group. This could indicate a resistance-associated fitness cost in the absence of selection. For example, insecticide resistance gene presence has been shown to confer reductions in adult insects’ size, longevity, and fecundity (Kliot and Ghanim 2012). As a consequence of the cost, reversions of resistance occur frequently in colonized populations (Freeman et al. 2021). We observed a large amount of variance and plasticity for both *Cx. restuans* field populations, but not as much for the *Cx. pipiens* from the colony. Laboratory populations consistently reared under standardized conditions can retain reduced genetic variation for some characteristics (Schneider et al. 2011). Our colony of *Cx. pipiens* showed reduced variation in responses to insecticide exposure compared to its original establishment, with only 28.5% mortality in the parental generation’s bottle bioassays (unpublished data). In the current experiment, the *Cx. pipiens* rarely survived until the diagnostic time, but there is still a significant genotype effect. There was a significant environmental effect on the wing size of the *Cx. pipiens* colony and the *Cx. restuans* from southern IL, but not for those from central IL. There was, however, a positive relationship between wing size and resistance for the central IL *Cx. restuans*. Another study found that larger *Anopheles* mosquitoes were more likely to survive insecticide treatment, which was attributed to the available nutritional reserves (Owusu et al. 2017).

The different feeding regimes in the two experiments make direct species comparisons challenging. However, both species exhibited similar trends: shorter wing lengths in the low food treatment and longer wing lengths in the high food treatment, despite differing reaction norms for resistance. While the feeding differences may have influenced the results, they do not affect our main conclusions about plasticity in resistance. Additionally, we recognize that the energetic reserves provided by the treatments may differ from those obtained solely from the larval diet, largely because of a shared adult environment that included access to a honey solution (i.e., sugar). However, a recent study indicates that while sugars provide enough energy for longevity, they may not satisfy the energy requirements for the rapid detoxification of insecticides (Cissé-Niambélé et al. 2025). This suggests that sugar feeding during the adult stage likely did not have a significant impact on the results.

The finding that wing size correlated with resistance in the central IL *Cx. restuans* population, but not the other populations, indicates different physiological resistance mechanisms may be involved. Most computational models simulating insecticide resistance management treat resistance as a monogenic trait linked to single gene mutations (Consortium 2010), although genome-wide association studies show that insecticide resistance in mosquitoes is highly polygenic (Lucas et al. 2023). However, insecticide resistance in mosquitoes is likely a mix of both monogenic and polygenic influences (Hobbs and Hastings 2025). Previous work found multi-function oxidases and α-esterase as significant insecticide detoxification mechanisms in IL *Cx. restuans* populations, while resistance in *Cx. pipiens* was also associated with multi-function oxidases, and a point mutation conferring knockdown resistance (Noel et al. 2025). Wood et al. (Wood et al. 2010) suggest cuticular thickening is likely to be selected along with monooxygenase-based resistance as it slows or regulates insecticide uptake, giving time to produce sufficient enzymes to catalyze pyrethroid metabolism. Metabolic traits, including those related to insecticide detoxification, can exhibit significant plasticity in response to changes in an animal’s internal state or environment. This phenotypic plasticity is context-dependent and can vary within and among individuals and different populations (Fox et al. 2018). Thus, the differences between the populations could potentially be attributed to plasticity in metabolic rate. For the *Cx. pipiens*, the lack of difference in resistance levels between food treatments could be from the colony losing the knockdown resistance allele over time. This highlights the need for further research into the resistance mechanisms present in *Cx. restuans* and their interactions with environmental conditions.

The H^2^ values for insecticide resistance provide evidence that mosquito genotype affects resistance, as hypothesized. It is important to note that these estimates are broad-sense measures and are likely inflated (Falconer 1996), though similarly high measures have been detected previously (Chan and Zairi 2013). Stressful conditions can increase the heritability of a trait under direct selection (Hoffmann et al. 1999), and this pattern has been observed in *Drosophila melanogaster* exposed to insecticide and temperature stress (Fournier-Level et al. 2016; Young et al. 2020). Nutritional stress might be the cause of the *Cx. restuans* from central IL having a higher heritability value in the nutrient-poor environment. Additionally, environmental stressors can induce heritable epigenetic modifications, which affect the organisms’ phenotypic responses to their environment by reprogramming gene expression (Mogilicherla and Roy 2023). Thus, another hypothesis is that the higher heritability under nutrient-poor conditions could also be due to heritable epigenetic modifications.

Interestingly, the southern IL *Cx. restuans* had a higher insecticide resistance heritability value in the high food treatment group, which displayed lower average resistance. This indicates that the nutrient-rich environment could represent stressful conditions for this group or that they are better suited for their home environment. Local adaptation, for example, is the process by which populations develop traits that enhance their survival and reproduction in specific environments, driven by the alignment between adaptive genetic variation and environmental conditions (Blanquart et al. 2013). The southern *Cx. restuans* population may be exhibiting local adaptation to lower food availability or higher densities, which could make a high food treatment a more toxic or stressful environment, though additional testing would be required to confirm a true local adaptation (i.e., reciprocal transplants). A toxic environment could occur from nitrogenous waste buildup associated with protein metabolism, making the environment more stressful (van Schoor et al. 2020), compared to their natural environment. Introducing organisms to novel environmental conditions can increase levels of heritable genetic variation and, subsequently, heritability estimates (Holloway et al. 1990). If the southern IL population of *Cx. restuans* have never been exposed to an environment like that created by the high food treatment, it makes sense to see that group’s high heritability estimate.

Insecticide resistance in *Cx. restuans* was influenced by the interaction between food treatment and collection location. Inhabiting different macroenvironments with different selection pressures can lead to phenotypic and genotypic differentiation among populations (Conner and Hartl 2004). Such regional differences have been shown for *An. gambiae*, where mosquitoes from coastal regions have higher tolerance to salt concentrations, while urban populations were found to be highly resistant to insecticides and environmental pollutants (Kamdem et al. 2017). Additionally, variations in microbiome composition have been shown to correlate with detoxification gene expression and insecticide resistance, indicating environmental differences that change the microbiome composition can also shape a population’s resistance level (Zhang et al. 2023; Malook et al. 2025). Given this, the opposing resistance responses between collection locations could be attributed to differences in insecticidal selection pressures, environmental pollution levels, or microbiota. When looking at the *Cx. pipiens* results, the importance of the collection location environment becomes apparent. The colony mosquitoes have been reared under optimal conditions and are thus not under any selective pressures related to insecticides, and the resulting phenotypic response average remains similar between the two diet treatments.

From an applied perspective, larval food availability significantly influenced the insecticide resistance level measured by the bioassay, and this effect varied across populations from different geographic regions. One important caveat to consider is that while the CDC bottle bioassay is designed to monitor the emergence and spread of insecticide resistance, one of its major limitations is that it does not translate directly to field efficacy. However, determining the field efficacy of permethrin is outside of the scope of this paper. Our results confirm that while carefully controlling food quantity and larval densities is essential for comparing and interpreting assay results, the observed G×E interactions suggest it may be beneficial to conduct assays across a food level range that reflects the variation mosquito larvae experience in the field. Further, the heritability levels and the range of outcomes observed across individual families highlight that the selection of specimens used in bottle bioassays can likewise introduce a large amount of variation. So, if a location’s resistance monitoring is based on collections of relatively few egg rafts, the measured resistance level could significantly deviate from the true population mean. An ideal sampling scheme might use a single female per egg raft to contribute to the bioassay. Additionally, detailed reporting of lab colony maintenance and history of field-sampled mosquitoes is needed. We suspect that considering these issues could help improve bottle bioassay reliability and provide a clearer link to implications for resistance under field conditions.

These findings have significant implications for defining resistance and evaluating current methods for testing resistance levels. Our findings suggest that caution should be used when comparing laboratory-colonized mosquitoes and field populations, as laboratory-reared populations can lose genetic diversity over time. Since laboratory colonies are frequently used to address key evolutionary questions, further work is needed to develop methods ensuring their genetic diversity reflects natural populations. Additionally, the local adaptive signatures between *Cx. restuans* populations reinforce the need for frequent, fine-scale resistance monitoring and indicate that population-specific data should inform vector control schemes. Local adaptation can significantly influence disease dynamics as vectors adjust to their environment and acquire new traits, potentially increasing the arboviral threat to humans. Although this study demonstrates the evolutionary potential for pyrethroid resistance, further research using mosquito populations with known resistance mechanisms is needed to determine whether heritability rates and phenotypic plasticity vary across specific mechanisms, as some mechanisms may be more influenced by environmental conditions or come with higher fitness costs than others.

## Supplementary information

Supplementary File 1

## Data Availability

The datasets used and/or analyzed during the current study are available from the University of Illinois Urbana-Champaign IDEALS data repository (https://hdl.handle.net/2142/128891).
