# Supplementary material for: Genotype by environmental interactions shape insecticide resistance phenotypes in Culex pipiens and Culex restuans
Source: Heredity (Edinb). 2026 Mar 7;135(5):313–22. doi: 10.1038/s41437-026-00833-w (PMC13219635; doi:10.1038/s41437-026-00833-w)
Supplement: Supplementary file 1 [file 41437_2026_833_MOESM1_ESM.docx]

**Table S1.** GPS coordinates for the sampling locations for the *Culex restuans* egg rafts in Illinois.

| **Location** | **Latitude** | **Longitude** |
| --- | --- | --- |
| Metropolis | 37.1427 | -88.6751 |
| Urbana | 40.1030 | -88.1938 |

**Table S2.** GPS coordinates for the egg raft sampling locations in Illinois, which were combined and used to establish the *Culex pipiens* colony.

| **Location** | **Latitude** | **Longitude** |
| --- | --- | --- |
| Mustang | 38.8679 | -89.8981 |
| Mimosa | 38.8482 | -90.0613 |
| Pontoon | 38.7192 | -90.0751 |
| Vivian | 38.6816 | -90.0084 |
